# Overexpression of c-Myc triggers p62 aggregation-mediated mitochondrial mitophagy in cabozantinib resistance of hepatocellular carcinoma

**DOI:** 10.1186/s10020-025-01263-w

**Published:** 2025-05-27

**Authors:** Kaibo Yang, Xing Zhang, Kun Yang, Sinan Liu, Jingyao Zhang, Yunong Fu, Tong Liu, Kunjin Wu, Jing Li, Chang Liu, Qichao Huang, Kai Qu

**Affiliations:** 1 Department of Hepatobiliary Surgery and Liver Transplantation, The Second Affiliated Hospital of Xi’an Jiaotong University, Xi’an, 710004 China; 2 Key Laboratory of Surgical Critical Care and Life Support (Xi’an Jiaotong University), Ministry of Education, Xi’an, 710004 China; 3 State Key Laboratory of Holistic Integrative Management of Gastrointestinal Cancers, Department of Physiology and Pathophysiology, Fourth Military Medical University, Xi’an, 710032 China; 4 Department of Surgical Intensive Care Unit, First Affiliated Hospital of Xi’an Jiaotong University, Xi’an, 710061 China

**Keywords:** HCC, Cabozantinib resistance, C-Myc, P62 aggregation, Mitophagy

## Abstract

Resistance to tyrosine kinase inhibitors (TKIs) poses a significant challenge in the treatment of hepatocellular carcinoma (HCC). Although dysregulation of mitochondrial dynamics has been implicated in the aggressive behaviors of various tumors, the specific role and underlying mechanisms by which this dysregulation contributes to cabozantinib resistance in HCC cells remains insufficiently characterized. By investigating mitochondrial dynamics as central regulators of cabozantinib resistance, this work specifically aims to discover actionable targets for restoring drug sensitivity in treatment-refractory HCC cells. We employed transmission electron microscopy (TEM) and confocal microscopy to analyze mitochondrial morphology in HCC cells resistant to TKIs. Additionally, we utilized an oncogene hydrodynamic injection-induced primary liver cancer mouse model to assess the therapeutic efficacy of combining cabozantinib with other pharmacological agents. Our results demonstrated significant increases in mitochondrial fragmentation, p62 aggregation, and mitophagy in cabozantinib-resistant HCC cells, which correlated with overexpression of c-Myc. Notably, inhibiting mitochondrial fission, p62 aggregation, or autophagy effectively reversed the resistance of HCC cells to cabozantinib. Mechanistically, cabozantinib treatment was shown to induce c-Myc expression, which significantly enhanced mitochondrial fragmentation and p62 aggregation, thereby promoting mitophagy. This mitophagic process selectively eliminated damaged mitochondria, reducing cytochrome C-induced apoptosis in cabozantinib-resistant cells. Ultimately, combining cabozantinib with either the autophagy inhibitor chloroquine or the p62 aggregation inhibitor XRK3F2 resulted in improved anticancer efficacy. In conclusion, c-Myc overexpression facilitates p62 aggregation-mediated mitophagy, leading to cabozantinib resistance in HCC cells. Inhibition of autophagy effectively restores cabozantinib sensitivity in HCC.

**Supplementary Information:**

The online version contains supplementary material available at 10.1186/s10020-025-01263-w.

## Introduction

Primary liver cancer is the third leading cause of cancer death, with hepatocellular carcinoma accounting for 80% of cases (Sung et al. 2021; Zhou et al. 2019). Targeted therapy provides options for advanced HCC (Zhou et al. 2023). The c-MET pathway is one of therapeutic targets in targeted therapy, and it has been established that this pathway plays a critical role in promoting the malignant phenotype of liver cancer, with nearly 30% of HCC patients exhibiting overexpression of c-MET (Lee et al. 2013), blocking this pathway can effectively inhibit the growth of liver cancer (You et al. 2011). Furthermore, activation of the c-MET pathway is a common mechanism of resistance in cancer treatment. Consequently, reversing liver cancer resistance by targeting the c-MET pathway has shown promising results, cabozantinib is a notable success in this regard (Abou-Alfa et al. 2018). In the CELESTIAL trial, cabozantinib exhibited a statistically significant enhancement in overall survival compared to placebo among HCC patients resistant to sorafenib However, the c-MET pathway often engages in cross-talk with other signaling pathways, making c-MET inhibitors such as cabozantinib susceptible to acquired resistance. Therefore, current researchers are inclined to investigate the combination of cabozantinib with other agents to mitigate the risk of acquired resistance (Zhao et al. 2021).

Preclinical investigations have demonstrated that cabozantinib primarily impedes HCC growth through mediation of the c-Met pathway (Shang et al. 2021). C-Met, a receptor for hepatocyte growth factor (HGF), experiences overexpression in up to 40% of HCC patients and is localized both on the cytomembrane and mitochondria (Guo et al. 2010; Yakes et al. 2011). Prior studies have indicated that c-Met signaling significantly influences mitochondrial physiology (Zhang et al. 2020). Blocking c-Met signaling disrupts mitochondrial function and incurs hepatocyte damage within hepatic cells (Gomez-Quiroz et al. 2016). Maintenance of mitochondrial function crucially relies on mitochondrial dynamics encompassing fusion, fission, and mitophagy (Eisner et al. 2018). Mitochondrial fission contributes to quality control, while mitophagy eliminates impaired mitochondria during cellular stress (Youle and Bliek 2012). Mitophagy has been validated as a mechanism that enhances the responsiveness of HCC cells to sorafenib treatment (Ma et al. 2023). However, the influence of mitochondrial morphology and dynamics on the effectiveness of cabozantinib remains unclear.

Mitophagy is a special form of autophagy that selectively removes dysfunctional mitochondria, ensuring the maintenance of cell function. It exerts significant and diverse functions by facilitating the clearance of redundant metabolic byproducts, thereby eliminating various free radicals released by these entities (Palikaras et al. 2018). Consequently, this mechanism safeguards cells against excessive reactive oxygen species (ROS) generation, ensuring the preservation of normal cellular physiology and functionality (Ren et al. 2023). Recent investigations have shed light on the critical involvement of p62 aggregation and phase separation in the initiation and progression of autophagy (Lei et al. 2021; Peng et al. 2021; Sun et al. 2018). Notably, report has demonstrated that polyubiquitin chain-mediated p62 phase separation effectively triggers autophagic initiation within cellular context (Sun et al. 2018). Additionally, concurrent observations have unveiled an interaction between ubiquitylated NUR77 on mitochondria and the UBA domain of p62, ultimately culminating in mitophagy induction (Peng et al. 2021). These findings suggest that the activation of mitophagy plays a role in tumor cells adapting to harsh environments, with p62 phase separation being involved in this process.

In this study, we established an acquired resistance model to cabozantinib utilizing cell cultures and discovered that augmented mitophagy contributes to drug resistance. Mechanistically, we demonstrated that c-Myc, previously reported to confer resistance against c-Met inhibitors, promotes p62 aggregation and propels mitophagy within cabozantinib-resistant cells. Furthermore, disruption of both mitophagy and p62 aggregation restored drug sensitivity in these resistant cell populations. Our investigation has solidified the fundamental involvement of mitophagy in cabozantinib resistance and posits that inhibiting mitophagy synergistically augments the efficacy of cabozantinib.

## Materials and methods

### Cell lines

Human HCC cell lines SNU-368 and SNU-739 were obtained from the Shanghai Cell Bank of the Chinese Academy of Sciences (Shanghai, China). Tumor cells were cultured in Dulbecco’s modified eagle’s medium (DMEM, Gibco, Grand Island, NY, USA) supplemented with 10% fetal bovine serum (FBS, Gibco, Grand Island, NY, USA). This study was approved by the Ethics Committee of Medical College of Xi’an Jiaotong University.

### Construction of cabozantinib-resistant HCC cells

To establish cabozantinib-resistant cells. SNU-368 and SNU-739 cells were treated with 0.25 µM cabozantinib, and increased the dose of 0.25 µM per week. After 5 months, we obtained the cabozantinib-resistant SNU-368 and SNU-739 cells. SNU-368-R and SNU-739-R cells were cultured in 5µM cabozantinib to maintain drug resistance.

### Knockdown, force expression of target genes

The c-Myc overexpression plasmids and empty vectors were purchased from Addgene. For transfection, A density of 2 × 10^5^ cells cultured in six-well plates. Then, the vectors were respectively transfected into cells by lipofectamine 2000(Invitrogen, USA). The transfection of siRNAs was conducted using Lipofectamine™ 2000(Invitrogen, USA) according to the manufacturer’s protocol. After 48 h infection the cells were selected with G418 (DIYBio) for 2 weeks to screen for stable transfected cells. C-MYC and PAKN siRNA were purchased from GenePharma (Shanghai, China), sequences were as followed.

- C-MYC: guide (5’→3’) UUAGAACAGCAAUAGCAUCCU, passenger (5’→3’) GAUGCUAUUGCUGUUCUAAUU
- C-MYC: guide (5’→3’) UUAGAACAGCAAUAGCAUCCU, passenger (5’→3’) GAUGCUAUUGCUGUUCUAAUU

### Antibody and reagent

The primary antibodies used in this study and their working concentration were showed in table. Cabozantinib, Carbonyl cyanide 3-chlorophenylhydrazone (CCCP), and Carbonyl cyanide 4-(trifluoromethoxy) phenylhydrazone (FCCP) were purchased from Meilunbio-Dalian (MB1666, MB2524, and MB3642).

**Table:** 

Antibody	Company (Cat. No.)	Working dilutions
c-Myc	proteintech(10828-1-AP)	1:5000
Cleaved Caspase3	Abcam(ab32042)	1:500
CYTC	immunoway(YM3402)	1:1000
PARKIN	proteintech(14060-1-AP)	1:500
PINK1	proteintech(23274-1-AP)	1:500
LC3-II	proteintech(18725-1-AP)	1:300
LC3	proteintech(14600-1-AP)	1:2000
SQSTM1	proteintech(18420)	1:1000
COX4	proteintech (11242-1-AP)	1:8000
DRP1	proteintech (2957-1-AP)	1:4000
DRP1 pS616	CST (#4494)	1:1000
DRP1 pS637	CST (#4867)	1:1000
MFN1	proteintech (13798-1-AP)	1:5000
MFN2	proteintech (12186-1-AP)	1:10000
PGC1α	proteintech (66369-1-Ig)	1:8000
BAX	proteintech (50599-2-Ig)	1:4000
BCL2	proteintech (12789-1-AP)	1:5000
Cleaved caspase9	CST (#9509)	1:1000
TOM20	proteintech (11802-1-AP)	1:200
β-actin	abways(AB0035)	1:3000

### Western blot

The cells were lysed with RIPA lysate (HAT, Xi’an, China), with protease inhibitors and PMSF (HAT, Xi’an, China). Protein from different cells were separated by 10% or 15% sodium dodecyl sulphate polyacrylamide gel and then transferred to a polyvinylidene fluoride (PVDF) membrane (Invitrogen, USA) according to the standard process. Then, the PVDF membranes were blocked by 10% non-fat milk in PBST for 2 h. The PVDF membranes were incubated overnight at 4 °C with primary antibodies and secondary antibody for one hour at 37℃. Beta-actin was the internal reference protein of all western blot. The proteins were visualized using ECL chemiluminescent substrate (NCM, Suzhou, China) and The Bio-Rad Image Lab software. (Bio-Rad, Hercules, CA, USA)

### Transmission electron microscopy

Cells were washed with 0.1 cacodylate buffer (pH 7.4) and fixed in electron microscope fixative (2% paraformaldehyde, 2.5% glutaraldehyde, and 0.1 mol/L sodium cacodylate). According to the standard protocols, prepared the thin sections and stained with uranyl acetate and lead citrate. Samples were observed by Leica transmission electron microscope.

### MT-keima dual color fluorescent probe analysis

Cells were infected with mt-Keima adenovirus (Hanheng Biotechnology) at optimized MOI. For adherent cells, half-volume infection was performed (4 h incubation followed by medium replenishment). After 36–48 h, dual-excitation imaging (440 nm [neutral pH, green] and 550 nm [acidic pH, red], emission 620 nm) was conducted using a confocal microscope (Leica).

### Immunohistochemistry

Immunohistochemical analysis of paraffin sections was performed using an IHC test kit (ZSGB-BIO, Beijing, China) according to the manufacturer’s instructions. The tumor tissues of mice were collected and fixed with 10% formalin, paraffin embedded tissue sections were dewaxed with xylene, and then hydrated with a series of gradient ethanol. Trisodium citrate 3 g and citric acid 0.4 g were dissolved in 1000 mL double distilled water to prepare antigen repair solution. After antigen repair, endogenous peroxidase blocker was used to block endogenous peroxidase. Then, blocking the non-specific binding site by the goat serum, tissue sections was incubated with primary and secondary antibodies. DAB was used for color development and hematoxylin was used for re-dyeing. After gradient ethanol dehydration and xylene transparent treatment, the sheet was sealed with neutral resin.

### Cell viability assay (Trypan blue exclusion method)

After treatment, cells were harvested and resuspended to obtain a single-cell suspension. An equal volume of 0.4% trypan blue solution was mixed with the cell suspension and incubated at room temperature for 1–3 min. Viable cells, which exclude the dye, were counted using a hemocytometer under a light microscope. Cell viability was calculated as the percentage of viable cells relative to the total number of cells, reflecting the survival rate after treatment.

### JC-1 mitochondrial membrane potential assay

Cells grown on glass-bottom dishes were stained with JC-1 (5 µg/mL, Beyotime) at 37 °C in the dark for 20 min. After washing with PBS, live cells were immediately imaged using a confocal microscope (Leica TCS SP8) with dual-channel detection: 488/530 nm (green monomeric form) and 543/590 nm (red J-aggregates). The membrane potential was assessed using ImageJ software (NIH, Bethesda, MD) and expressed as the red/green fluorescence intensity ratio.

### Immunofluorescence

Tumor tissues from mice were fixed in 4% paraformaldehyde (PFA), embedded in paraffin, and sectioned at 4-µm thickness, followed by dewaxing with xylene and rehydration through a graded ethanol series. For tissue sections, antigen retrieval was conducted by heating slides in citrate buffer (pH 6.0) and cooling to room temperature. Cultured cells were fixed with 4% PFA for 15 min and permeabilized with 0.1% Triton X-100 in PBS for 10 min. Both cell and tissue samples were blocked with 10% goat serum for 1 h at room temperature, incubated with primary antibodies (1:200 dilution in Antibody Diluent) overnight at 4 °C, washed with PBS, and then treated with fluorescence-conjugated secondary antibodies (Alexa Fluor 488/633, 1:200) for 1 h in the dark. Nuclei were stained with DAPI (5 µg/mL, 5 min), and slides were mounted with anti-fade mounting medium. Images were captured using a confocal microscope (Zeiss LSM 880, Germany) and analyzed with ImageJ software.

### Mitochondrial network imaging by electron microscopy and confocal microscopy

The cells were fixed by the addition of 2.5% glutaraldehyde, Then the cells were rinsed with PBS buffer and post-fixed using OsO4 for 1 h. After alcohol dehydrated and embedded in epoxy resin. A diamond knife was used to cut Ultra-thin Sects. (70–100 nm). Next, the sections were stained by 5% uranyl acetate in 50% ethanol and 2% aqueous lead citrate solution. Tecnai G2 electron microscope (FEI, cHillsboro, Oregon) was performed to analyze the sections, at 11,500 magnifications. The Mito-Tracker Red CMXRos (Beyotime, Shanghai, China) and Mito-Tracker Green (Beyotime, Shanghai, China) were used to observe mitochondrial morphology in viable cells. Then, cells were monitor by an Olympus FV 1000 laser-scanning confocal microscope (Olympus Corporation, Tokyo, Japan). The ImageJ software (NIH, Bethesda, MD) was used to measure mitochondria for morphometric analysis.

### Mitochondrial morphology and damage quantification

Mitochondrial morphology in individual cells into three categories: Elongated (≥ 75% mitochondria exhibiting elongated morphology), intermediate (25–75% elongated mitochondria), and fragmented (≤ 25% elongated mitochondria). Elongated morphology was defined as mitochondrial structures exceeding 1 μm in length. mitochondrial damage was assessed and quantified based on ultrastructural changes observed by transmission electron microscopy. Mitochondria were classified as either healthy or damaged. Healthy mitochondria were defined as those with intact double membranes, organized cristae, and dense matrix. Damaged mitochondria were characterized by partial or complete cristae disassembly, matrix swelling, and/or outer membrane rupture (Chakraborty et al. 2020). The number of healthy and damaged mitochondria were counted, and the proportion of damaged mitochondria was calculated and compared between different experimental groups. Two independent blinded investigators performed the above evaluation, with final categorizations determined by consensus.

### Nude mice xenograft model

Animal experiments have been approved by the animal ethics committee of Xi’an Jiaotong University. Four-week-old Male BALB/c nude mice were ordered from the Medical Experimental Animal Center, Xi’an Jiaotong University. The mice were weight of 16 to 20 g and randomly divided into groups(*n* = 30): (1) control group (2) c-Myc overexpression (3) cabozantinib (4) cabozantinib + mdivi1(5) cabozantinib + XRK3 F2. For the construction of xenograft tumor model, 10^7^ SNU-368 were injected into the left flank of the mice. When the tumor diameter reached 2–3 mm. The same amount of Dimethyl Sulfoxide (BIOTAL, Shanghai, China) was used as the control treatment. After one month, the mice were sacrificed and the tumors were collected.

### Cell viability and apoptosis assays

The cell counting kit-8 (CCK-8) assay (7 seabiotech, Shanghai, China) was performed to detected the Cell viability. Absorbance values at 490 nm wavelength were measured using a microplate reader (Bio-Tek, Winooski, VT, USA). Annexin V-PE and 7 AAD Apoptosis Detection Kit (BD, USA) was used to determine the cell apoptosis. The frequency of apoptotic cells was analyzed with flow cytometry (Beckman Coulter, Fullerton, CA, USA). The cell apoptosis was also assessed with Caspase 3/7 Activity Apoptosis Assay Kit (Sangon Biotech, Shanghai, China). All experiments followed the manufacturer’s instructions.

### Evaluation of fluorescent LC3-II and mitochondria

Plasmid pcDNA3.1-GFP-LC3II was transfected into HCC cells with Lipofectamine™ 2000(Invitrogen, USA) and then stained with The Mito-Tracker Red CMXRos (Beyotime, Shanghai, China). The co-localization of LC3-II (green fluorescence) and mitochondria (red fluorescence) was observed by confocal microscopy.

### Hydrodynamic injection and mouse treatment

The plasmids pT3EF1a-c-Myc and pCMV/sleeping beauty transposase (SB) were purchased from Addgene. Male wild-type FVB/N mice were obtained from GemPharmatech (Nanjing, Jiangsu, China). SB-mediated hydrodynamic injection was performed as previously described. Cabozantinib (MedChemExpress), XRK3 F2 (MedChemExpress) and CQ (MedChemExpress) were intraperitoneally injected into mice.

### Statistical analysis

R 4.0.2, R Studio 2.1, SPSS 21.0 software (IBM Corp., Armonk, NY, USA) and GraphPad Prism 8 (San Diego, CA, USA) were performed to analyze the data. In all statistical graphs, * denotes *p* < 0.05. Each group was quantified based on data from at least three independent experiments. In each experiment, at least 20 cells and 15 fields were quantified to ensure the reliability and repeatability of the results.

## Results

### Increased mitochondrial fragmentation in cabozantinib-resistant hepatocellular carcinoma cells

**Fig. 1:**
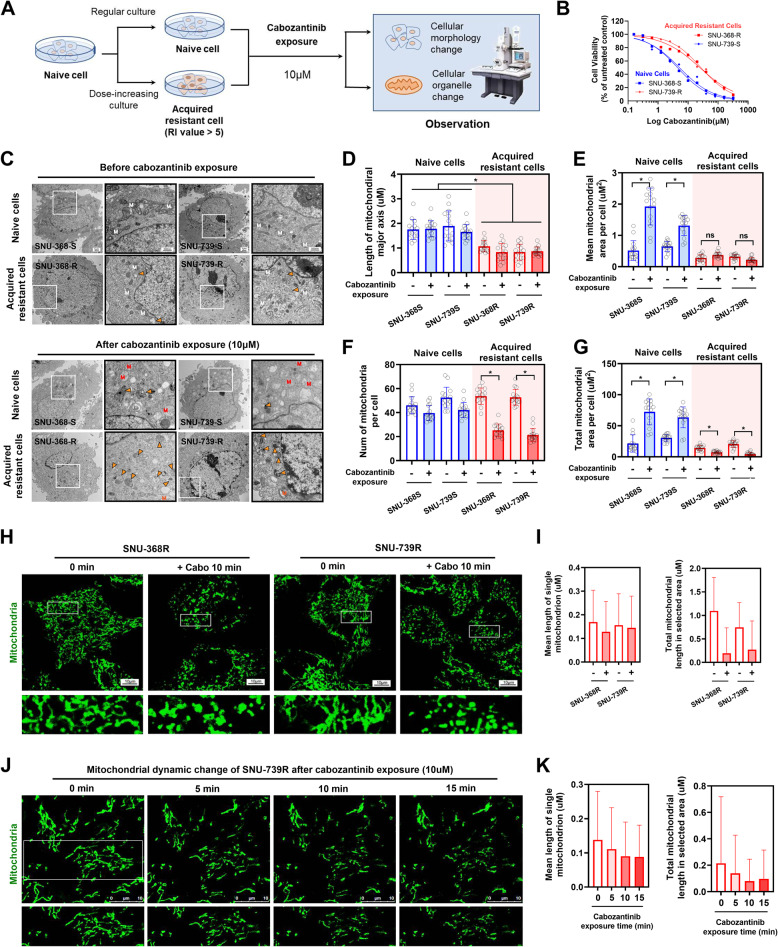
Increased Mitochondrial Fragmentation in Cabozantinib-Resistant HCC cells. **A** Schematic diagram of experimental workflow. **B** Dose-response analysis for SNU-368 and SNU-739 naive cells and their corresponding acquired resistant cells. The IC50 values for SNU-368-S, SNU-368-R were 4.45 µM and 24.80 µM, with a resistance factor of 5.57. For SNU-739-S, SNU-739-R, the IC50 values were 5.18 µM and 27.03 µM, with a resistance factor of 5.22. **C** Electron microscopy images showing the cellular structures of naive and acquired resistant cells. Cells were cultured under 10 µM cabozantinib pressure. White “M” represents normal mitochondria, red “M” represents damaged mitochondria, and solid arrows indicate autophagosomes. **D-G** Statistical analysis of electron microscopy results, including mitochondrial length, average area, count, and total area. **H-I** Confocal microscopy analysis of mitotracker labeled mitochondria in acquired resistant cells after cabozantinib treatment, focusing on mitochondrial morphology. Statistical parameters include individual mitochondrial average length and overall mitochondrial length within cells. **J-K** Time-lapse confocal microscopy observation of dynamic changes in mitochondrial morphology in acquired resistant cells treated with cabozantinib. Images were captured every 5 min with intervals to avoid fluorescence photobleaching. Statistical measures include individual mitochondrial average length and total mitochondrial length

Initially, screenings were carried out across several hepatocellular carcinoma (HCC) cell lines, leading to the identification of two cabozantinib-sensitive lines, SNU368 and SNU739 (Fig. 1A and FigS1). These cell lines were then subjected to gradient drug exposure to induce cabozantinib resistance, resulting in the development of resistant SNU368 and SNU739 cell lines, both exhibiting a resistance factor increase of approximately 5-fold (Fig. 1B). Notably, the resistant cells showed a significant reduction in mitochondrial size in comparison to the sensitive cells (Fig. 1C-E). Moreover, upon cabozantinib treatment, the resistant cell line exhibited substantially less mitochondrial damage than the sensitive line (Fig. 1C). Also, a significant reduction in both the quantity of mitochondria and the overall mitochondrial mass was noted in the resistant cells compared to the sensitive ones. (Fig. 1F-G). Mitochondrial visualization in live cells revealing a consistent pattern of mitochondrial fragmentation in the resistant cells following cabozantinib treatment, accompanied by a significant reduction in total mitochondrial mass (Fig. 1H-I). Furthermore, Time-lapse imaging analysis revealed rapid mitochondrial fragmentation in the resistant cells subsequent to cabozantinib treatment (Fig. 1J-K). Collectively, the results underscore that cabozantinib-resistant HCC cells have developed an enhanced capacity for rapid mitochondrial fragmentation.

### Increased mitophagy markedly enhances cabozantinib resistance in HCC cells

**Fig. 2:**
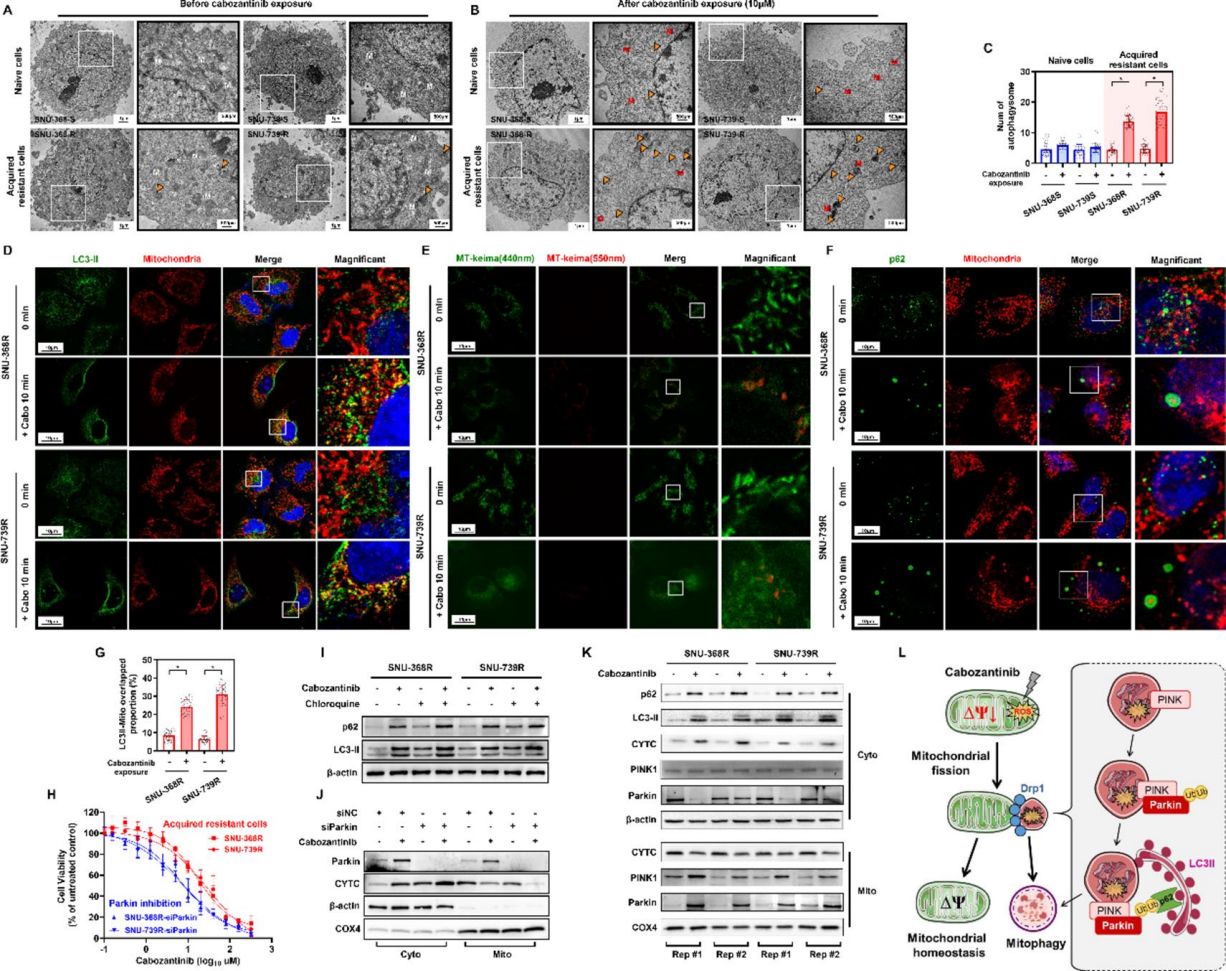
Increased Mitophagy Markedly Enhances Cabozantinib Resistance in HCC Cells. **A-B** Electron microscopy images showing the cellular structures of naive and acquired resistant cells. Cells were cultured under 10 µM cabozantinib pressure. White “M” represents normal mitochondria, red “M” represents damaged mitochondria, and solid arrows indicate autophagosomes. **C** TEM-based quantification of the number of autophagosomes in liver cancer cell lines with or without exposure to cabozantinib. **D** Confocal microscopy observation of LC3-II protein fluorescence and mitochondrial fluorescence in resistant cell lines with or without exposure to cabozantinib. Green indicates LC3-II protein, red indicates mitochondria, and blue represents DAPI. **E** MT-keima dual color fluorescent probe was used to evaluate the level of mitophagy in drug-resistant HCC cells treated with cabozantinib. The live cell imaging of MT-keima fluorescence (green: mitochondria at neutral pH; red: lysosomal phagocytosis of mitochondria at acidic pH) showed an increase in mitochondrial lysosome fusion in drug-resistant cells after treatment with cabozantinib. **F** Confocal microscopy observation of p62 protein fluorescence and mitochondrial fluorescence in resistant cells with or without exposure to cabozantinib. Green indicates p62 protein. **G** Quantification of the overlap between LC3-II protein fluorescence and mitochondrial fluorescence in resistant cell lines with or without exposure to cabozantinib. **H** Dose-response analysis in resistant cell lines after knocking down Parkin protein under cabozantinib stress. The IC50 of SNU-368-R decreased from 16.63 µM to 7.33 µM, and the IC50 of SNU-739-R decreased from 29.49 µM to 7.824 µM. **I** Western blot analysis of LC3II and P62. **J** Western blot analysis of Parkin and cytochrome C in cells and mitochondria after knocking down Parkin protein in cabozantinib-treated resistant cells. **K** Western blot analysis of mitochondrial-cytosolic partitioning for PINK1, Parkin, cytochrome C (CYTC), LC3-II and p62 in cabozantinib-treated resistant cells. Mitochondrial and cytosolic fractions were isolated via differential centrifugation prior to immunoblotting. Two technical replicates are shown for mitochondrial (Mito) and cytosolic (Cyto) fractions. **L** Proposed hypothesis for the mechanism of cabozantinib resistance in HCC cells

Mitophagy, an important mitochondrial quality control system, contributes to neoplastic progression and drug resistance in various types of tumors. The observed substantial rise in the number of autophagosomes, coupled with a reduction in mitochondrial mass in Cabozantinib-resistant cells relative to their Cabozantinib-sensitive counterparts (Fig. 1C and G, and Fig. 2A-C), suggests an augmentation of mitophagy in the Cabozantinib-resistant cells. Indeed, the co-localization of mitochondria with the LC3-II protein was found to be increased in drug-resistant cells following cabozantinib treatment (Fig. 2D and G). The results from the autophagy flux detection and the MT-keima dual-color fluorescent probe further confirmed the enhancement of mitochondrial autophagy (Fig. 2E and I and S2 A). Additionally, a pronounced aggregation of p62 was noted in the resistant cells after treatment with cabozantinib, which significantly co-localized with mitochondria (Fig. 2F). To assess the extent of mitophagy in drug-resistant cells, the expression of PINK1 and Parkin in mitochondria were further evaluated with and without cabozantinib treatment. Notably, an increase in mitochondria Parkin expression was detected post-Cabozantinib treatment in Cabozantinib-resistant HCC cells (Fig. 2K).

The effect of mitophagy on drug resistance was further investigated by silencing Parkin expression. As shown in Fig. 2F, an elevation in cellular Cytochrome C levels was detected in cells with Parkin knockdown post-Cabozantinib treatment, suggesting an increase in apoptosis. Consistently, a significant decrease in IC50 values was noted following the suppression of Parkin (Fig. 2H). In addition, conducted a more comprehensive characterization of mitochondrial dynamics and found that cabozantinib selectively enhances mitochondrial fission through specific upregulation of DRP1 phosphorylation at Serine 616 (DRP1 S616), while exhibiting negligible impacts on mitochondrial fusion machinery (MFN1/2) or biogenesis regulators (PGC-1α) (FigS2B). This finding suggests that drug resistance capabilities were compromised upon Parkin knockdown. Collectively, these results indicate that HCC cells may promote tumor drug resistance by augmenting mitophagy (Fig. 2L).

### c-Myc overexpression as a key mechanism driving cabozantinib resistance in HCC cells

**Fig. 3:**
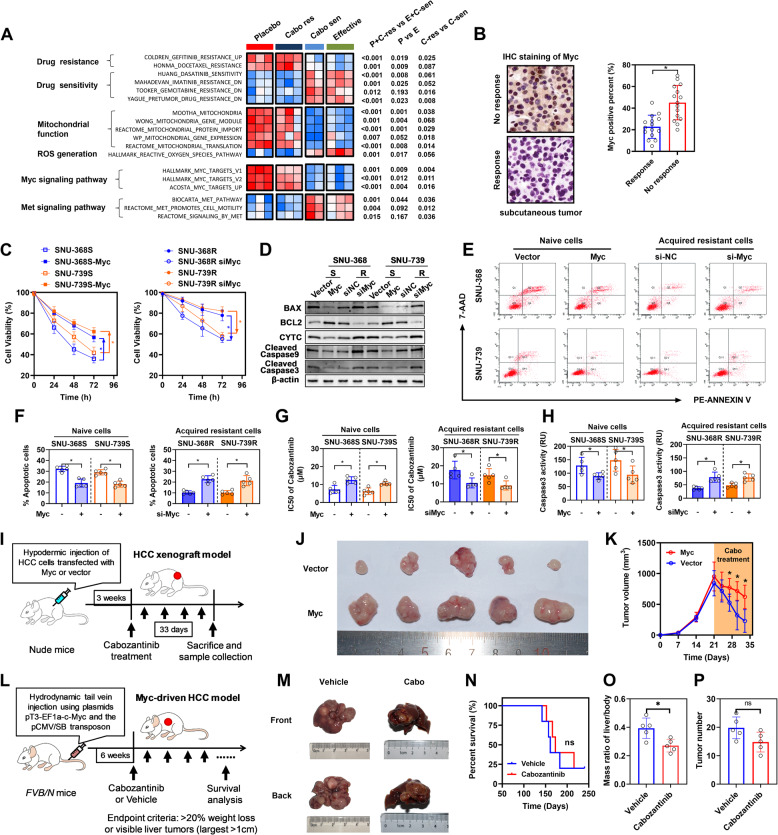
High Expression of c-Myc Promotes Cabozantinib Resistance in Liver Cancer Cells. **A** Differential expression pathway heatmap of cells with or without cabozantinib resistance (GSE174770). **B** Detection of c-Myc expression in subcutaneous tumor models established with resistant and sensitive liver cancer cell lines. **C** Cell viability assay using CCK-8 method after overexpressing c-Myc in cabozantinib-sensitive cells and knocking down c-Myc in resistant cells. **D** Western blot analysis of BAX, BCL2, CYTC, Cleaved caspase9 and Cleaved caspase3. **E-F** Apoptosis detection using flow cytometry after overexpressing c-Myc in cabozantinib-sensitive cells and knocking down c-Myc in resistant cells. **F** Dose-response analysis of cells after overexpressing c-Myc in cabozantinib-sensitive cells and knocking down c-Myc in resistant cells. The IC50 of SNU-368-S and SNU-739-S increased from 7.25 µM and 6.23 µM to 12.49 µM and 10.60 µM, respectively, while the IC50 of SNU-368-R and SNU-739-R decreased from 17.66 µM and 14.84 µM to 10.52 µM and 9.14 µM, respectively. **G** Caspase 3/7 Activity Apoptosis Assay to detect caspase3 activity in cells after overexpressing c-Myc in cabozantinib-sensitive cells and knocking down c-Myc in resistant cells. **H** Construction of subcutaneous tumor models in mice and administration schedule. **I-J** Images of subcutaneous tumor and changes in tumor volume. **K** Construction of c-Myc-induced orthotopic liver cancer model using the Sleeping Beauty system, **L** Gross images of the c-Myc-induced in situ liver cancer mouse model and its appearance after cabozantinib treatment. **M-O** Statistical analysis of mouse mortality rate, liver/body ratio and tumor number after cabozantinib treatment

**Fig. 4:**
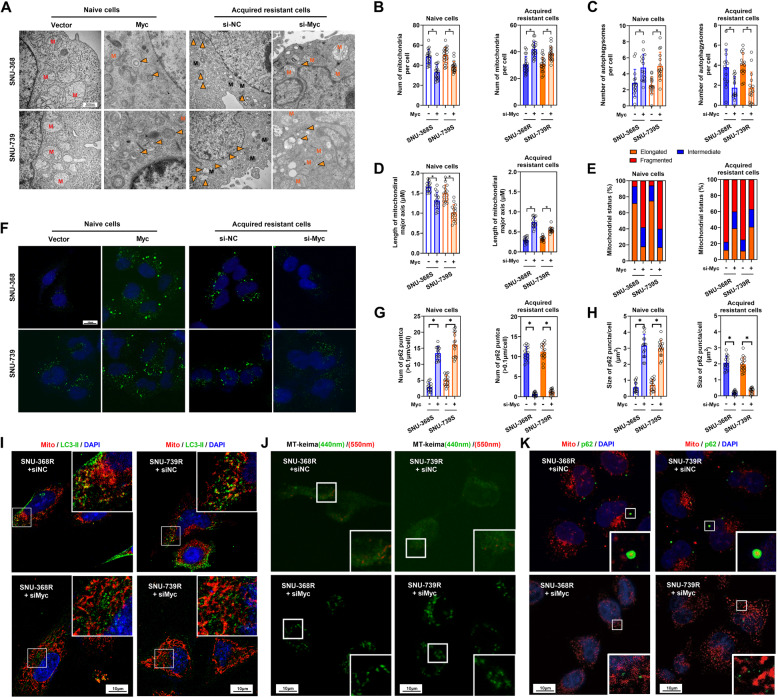
Expression of c-Myc Induces Mitochondrial Fragmentation and Mitophagy in HCC Cells. **A** Electron microscopy observation of mitochondrial damage and changes in the number of autophagosomes in cabozantinib-sensitive cells after c-Myc overexpression and in resistant cells after c-Myc knockdown. Cells were cultured under 10 µM cabozantinib stress. Black M represents normal mitochondria, red M represents severely damaged mitochondria, yellow M represents damaged mitochondria, and solid arrows indicate autophagosomes. **B-D** Quantification of mitochondrial number, autophagosome number, and mitochondrial major axis length in liver cancer cells under different treatments. **E** Statistical analysis of the proportion of different states of mitochondria in liver cancer cells. **F** Confocal microscopy observation of p62 protein. p62 protein is labeled in green and DAPI is shown in blue. **G-H** Statistical analysis of p62 puncta size and number. **I** Confocal microscopy observation of co-localization between LC3-II protein and mitochondria in liver cancer cells. LC3-II protein is labeled in green, mitochondria are labeled in red, and DAPI is shown in blue. **J** MT-keima dual color fluorescent probe was used to evaluate the level of mitophagy (green: mitochondria at neutral pH; red: lysosomal phagocytosis of mitochondria at acidic pH). **K** Confocal microscopy observation of co-localization between p62 protein and mitochondria in liver cancer cells. p62 protein is labeled in green, mitochondria are labeled in red, and DAPI is shown in blue

To further investigate the mechanisms contributing to drug resistance in HCC cells, we analyzed the transcriptional dataset GSE174770 from the GEO database. This dataset includes transcriptomic profiles of HCC cells treated with chemotherapy drugs, including placebo and cabozantinib monotherapy. Initially, all samples were classified into two distinct categories: the “no response” group and the “response” group, utilizing principal component analysis (PCA) for stratification. Notably, within the cabozantinib monotherapy group, three samples were classified as “no response,” while two were deemed as “response.” To validate the observed differences in tumor behavior, we employed single-sample gene set enrichment analysis (ssGSEA), uncovering significant behavioral disparities that supported our classification. Subsequent analysis of differentially expressed genes revealed marked variations in mitochondrial function and reduced reactive oxygen species (ROS) generation pathways in the “no response” group. Intriguingly, we observed pronounced alterations in c-Myc-associated pathways (Fig. 3A and FigS3). In parallel, our analysis of the GSE97098 dataset, containing IC50 values for cabozantinib across HCC cell lines, classified cell lines into resistant (IC50 ≥ 18 µM) and sensitive groups, revealing significantly elevated c-Myc expression in resistant cell lines (FigS4 A). Notably, this expression pattern was consistently observed in paired resistant/sensitive cell models (SNU-368 vs SNU-739), with resistant variants exhibiting heightened c-Myc protein levels relative to their sensitive counterparts (FigS4B), thereby confirming c-Myc ‘s functional involvement in therapeutic resistance.

To further explore these findings, we conducted subcutaneous tumorigenesis assays in murine models using sensitive and resistant cell lines, followed by immunohistochemical staining, which showed increased c-Myc expression in tumors from the resistant cell line (Fig. 3B). Cellular experiments involving c-Myc overexpression in sensitive cells and knockdown in resistant cells, under cabozantinib treatment, indicated that c-Myc expression levels significantly influence tumor cell mitochondrial membrane potential, viability and apoptosis (Fig. 3C-F and H and FigS4E-F). Moreover, assessing the IC50 for cabozantinib in HCC cells revealed that c-Myc overexpression increased resistance (Fig. 3G). To examine this effect in vivo, we established two animal models: one for subcutaneous tumor growth with varying levels of c-Myc expression and another employed a tail vein high-pressure c-Myc-plasmid injection to generate an orthotopic liver cancer model. The results demonstrated a significant reduction in the volume of subcutaneous tumors from wild-type cells compared to those with high c-Myc expression under cabozantinib treatment (Fig. 3I-K). Moreover, c-Myc-driven liver tumors exhibited inherent resistance to cabozantinib. Despite a modest reduction in the liver-to-body weight ratio observed in the cabozantinib-treated group, therapeutic efficacy was limited, as evidenced by the lack of significant differences in survival outcomes and tumor burden metrics between the cabozantinib and placebo groups (Fig. 3L-P). These findings collectively indicate that c-Myc upregulation is a key factor in the development of cabozantinib resistance in HCC cells.

### Expression of c-Myc induces mitochondrial fragmentation and mitophagy in HCC cells

To investigate the role of c-Myc in mitochondrial fragmentation in HCC cells, c-Myc overexpression was induced in SNU-368 S and SNU-739 S cells, while c-Myc knockdown was performed in SNU-368R and SNU-739R cells (FigS4 C). Subsequent analysis of mitochondrial morphology under cabozantinib-induced stress revealed that higher c-Myc expression levels led to increased mitochondrial fragmentation and a decrease in mitochondrial swelling and damage. Conversely, c-Myc knockdown produced the opposite effects (Fig. 4A-C and FigS4D). Quantitative evaluation further showed a significant decrease in the number of mitochondria, a reduction in mitochondrial size, and an elevation in mitophagosome numbers following c-Myc overexpression, with c-Myc knockdown having inverse outcomes (Fig. 4D-E). In addition, higher c-Myc expression levels lead to an increase in the number and size of P62 aggregates. On the contrary, c-Myc knockout produced the opposite effect (Fig. 4F-H). Immunofluorescence staining demonstrated that c-Myc knockdown in drug-resistant cells resulted in a more elongated mitochondrial phenotype, an absence of p62 aggregation, significantly reduced the MT-keima red fluorescence (550 nm) area and the co-localization between mitochondria and LC3-II protein (Fig. 4I-K). These findings suggest a notable decrease in mitophagy following c-Myc knockdown.

### p62 aggregation-mediated mitophagyplay a key role in drug resistance of HCC cells

**Fig. 5:**
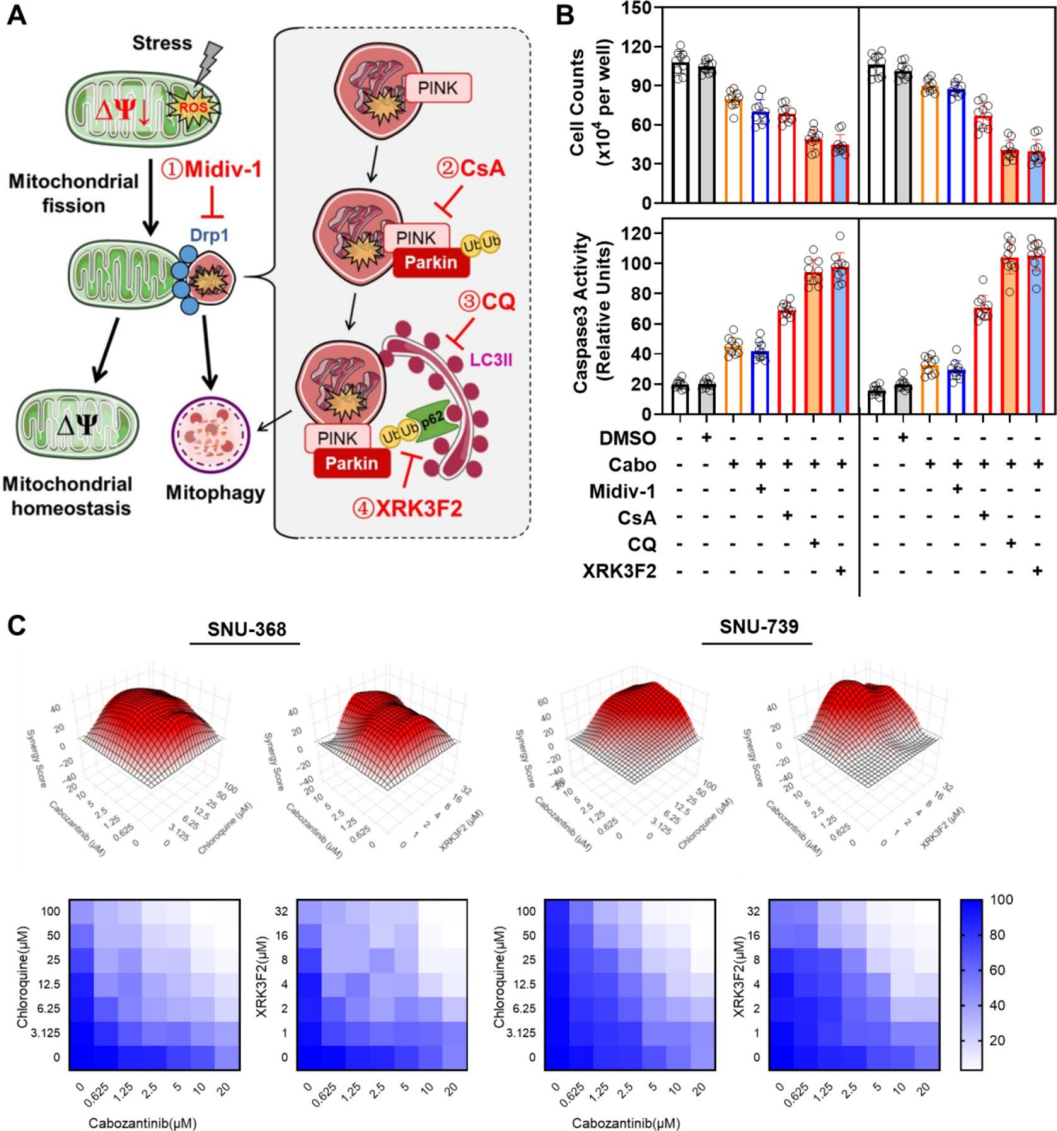
p62 Aggregation-Mediated Mitophagy Plays a Key Role in Drug Resistance of HCC Cells. **A** Schematic representation of the sites of action for each inhibitor. **B** Cell viability (live cells counted using hemocytometer after trypan blue exclusion) and intracellular Caspase3 activity (Caspase 3/7 Activity Apoptosis Assay) were assessed after treating cabozantinib-resistant HCC cells with different inhibitors. **C** Cell viability assay using CCK-8 method was evaluated in cabozantinib-resistant liver cancer cells following treatment with cabozantinib in combination with chloroquine and XRK3F2

Based on the aforementioned findings, we postulate that p62 aggregation-mediated mitophagy may play significant roles in drug resistance within HCC cells (Fig. 5A). To further elucidate this mechanism, we combined cabozantinib with several inhibitors separately to investigate HCC cell viability. Our data indicated that administration of cabozantinib with the mitochondrial fragmentation inhibitor, Midiv-1, led to a substantial reduction in tumor cell viability (Fig. 5B). Moreover, the combination of cabozantinib with autophagy inhibitors, such as cyclosporin A (CsA) or chloroquine, as well as the p62 aggregation inhibitor XRK3F2, resulted in a further pronounced reduction in cell viability, which was accompanied by a significant upregulation of Caspase3 activity (Fig. 5B). Drug synergy assays utilizing combinations of cabozantinib with chloroquine or XRK3F2 against resistant HCC cells revealed an enhanced cytotoxic effect (Fig. 5C). These findings collectively imply that p62 aggregation-mediated autophagy, specifically mitophagy, is actively involved in the development of resistance to cabozantinib in HCC cells. 

### Combination therapy enhances cabozantinib efficacy in c-Myc-driven orthotopic liver cancer

**Fig. 6:**
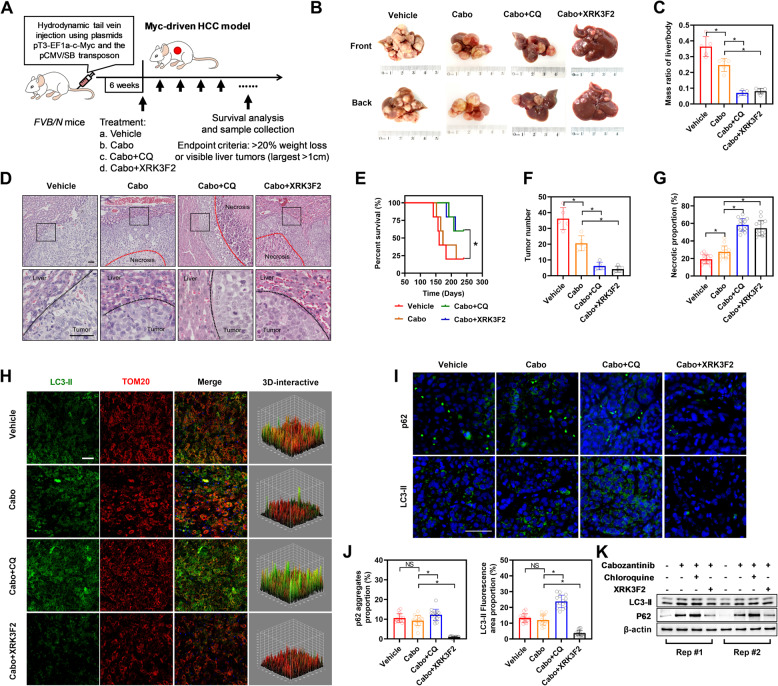
Combination Therapy Reverses Drug Resistance in c-Myc-Induced in Situ Liver Cancer Model. **A** Schematic diagram illustrating the construction of the c-Myc-induced in situ liver cancer mouse model and the drug administration process. **B** Gross images of the c-Myc-induced in situ liver cancer mouse model and its appearance after drug treatment. **C** Liver-to-body weight ratio in different groups of mice. **D** Hematoxylin and eosin (HE) staining images at the tumor margin in the livers of mice from each group. **E** Survival status of mice in each group. **F** Tumor number of mice in each group. **G** Percentage of necrotic area in liver cancer tissue of mice from each group. **H** Confocal microscopy observation of expression and colocalization of LC3-II protein with mitochondria in tissues of mice from each group. **I** Confocal microscopy observation of expression and colocalization of LC3-II and p62 proteins in tissues of mice from each group. **J** Percentage of aggregated p62 protein and percentage of LC3-II protein expression in tissues of mice from each group (calculated based on scanned area). **K** Western blot analysis of LC3-II, and p62 in tissues of mice from each group

To assess the effectiveness of combination therapy, a study was conducted using a c-Myc-driven orthotopic liver cancer mouse model (Fig. 6A). The results indicated that cabozantinib alone offered mild therapeutic benefits against c-Myc-induced primary liver cancer. However, its therapeutic efficacy was significantly enhanced when used in conjunction with either chloroquine or XRK3F2, leading to a marked increase in tumor suppression (Fig. 6B-C and F). Survival analyses further revealed that mice treated with the combination therapy exhibited a significantly extended survival period in comparison to those in the control group or those treated with cabozantinib alone (Fig. 6E). Morphological analysis of the dissected livers from the mice showed a notable increase in necrotic areas within tumors in the combination therapy group (Fig. 6D and G). Additionally, to investigate the extent of mitophagy within the tissues, fluorescence labeling and western blot was performed targeting LC3-II and p62 protein. Aligning with the observations from cell-based assays, the expression levels of LC3 and p62 proteins were elevated in the control group (characterized by high c-Myc expression), but their abundance decreased in the combination therapy group, indicating reduced mitophagy (Fig. 6H-K). These results suggest that while cabozantinib monotherapy possesses limited effectiveness in c-Myc-driven orthotopic liver cancer models, its combination with chloroquine or XRK3F2 markedly amplifies its anti-cancer properties. 

**Fig. 7:**
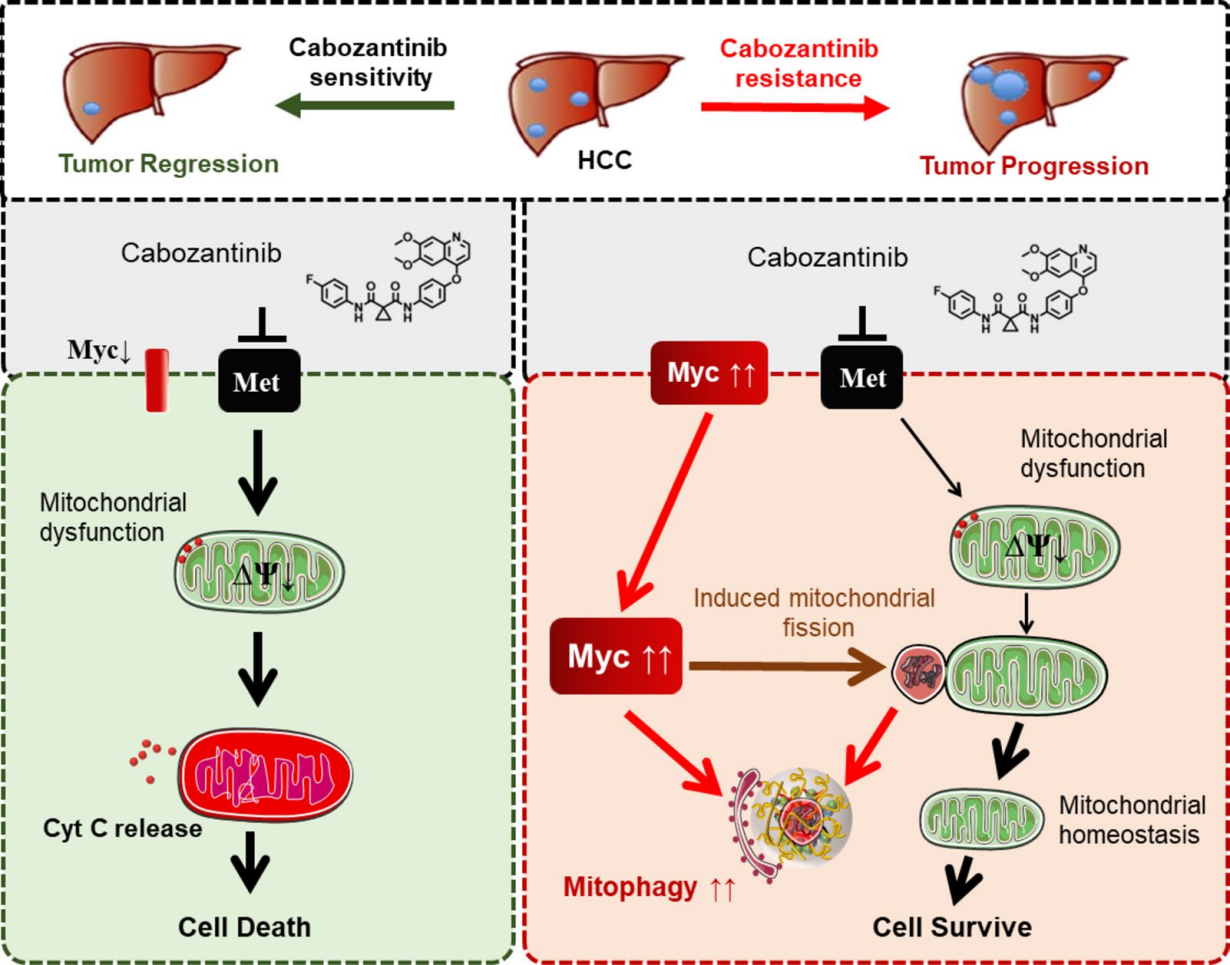
Hypothetical Mechanism Diagram. The results of this study suggest that in cabozantinib-resistant liver cancer cells, there is high expression of c-Myc. Elevated c-Myc expression promotes mitochondrial fission, p62 aggregation, and mitophagy, ultimately leading to the clearance of damaged mitochondria. This protective mechanism enhances tumor cell functionality and contributes to the development of cabozantinib resistance

Based on the collective evidence from mitochondrial dynamics, mitophagy, and c-Myc functional studies, we propose a unified mechanism underpinning cabozantinib resistance in HCC (Fig. 7). Elevated c-Myc expression orchestrates mitochondrial fragmentation via DRP1 activation, facilitates p62-aggregated mitophagy to eliminate damaged organelles, and ultimately sustains tumor cell survival under therapeutic stress.

## Discussion

Here, we investigated the characteristics of cabozantinib-resistant SNU-368 and SNU-739 cells, observing a significant augmentation of mitochondrial fission and mitophagy in these cells. Additionally, we discovered that p62 plays a pivotal role in this process. Through a series of in vitro and in vivo experiments, we identified enhanced p62 aggregation and mitophagy as essential mechanisms for reversing cabozantinib resistance in hepatocellular carcinoma cells. Consequently, targeting interventions aimed at promoting safe and effective modulation of p62 aggregation or mitochondrial dynamics represents a promising strategy to enhance cabozantinib therapy for liver cancer.

TKIs are critical components of comprehensive treatment for liver cancer (Haber et al. 2021). Despite recent advancements in immunotherapy, TKIs continue to be the preferred treatment option for most advanced liver cancer patients due to factors such as economic constraints, especially in low-resource regions (Allaire et al. 2022). However, both first-line drugs like sorafenib and lenvatinib, as well as second-line drugs like regorafenib and cabozantinib, face substantial challenges associated with drug resistance (Ladd et al. 2023). While research efforts have predominantly focused on understanding the resistance mechanisms of first-line drugs such as sorafenib and lenvatinib, less attention has been given to unraveling the resistance mechanisms of second-line drugs like cabozantinib. Exploring the mechanisms underlying cabozantinib resistance is of significant importance since patients often encounter a lack of effective treatment options when second-line drugs fail.

c-Myc, a well-established oncogene in hepatocellular carcinoma cells, has been implicated in promoting various malignant biological behaviors, including tumor development, drug resistance, and immune evasion (Liu et al. 2019; Wang et al. 2023). In c-Myc-induced liver cancer, the efficacy of cabozantinib monotherapy is limited (Shang et al. 2021). In our study, we observed alterations in multiple pathways, including c-Met, c-Myc, mitochondrial function, and ROS generation, in cabozantinib-resistant cells. c-Myc expression was significantly upregulated, and targeted interventions aimed at modulating c-Myc expression effectively reversed cabozantinib resistance in hepatocellular carcinoma cells. Furthermore, we observed limited efficacy of cabozantinib in c-Myc-induced spontaneous tumor mouse models, indicating that high c-Myc expression constitutes a key factor contributing to cabozantinib resistance in hepatocellular carcinoma cells. However, the precise impact of c-Myc on mitochondrial function, ROS clearance, and other biological processes remains to be elucidated.

Mitochondrial dynamics play a crucial role in maintaining cellular vitality and drug resistance in tumors (Sheng et al. 2019; Yu et al. 2021). As vital organelles within the cytoplasm, mitochondria are central to cellular activities. Stable maintenance of mitochondrial function is indispensable during the development of drug resistance in tumors. Enhanced mitochondrial biogenesis and mitophagy have been shown to promote tumor cell adaptation to adverse environments and contribute to drug resistance (Glytsou et al. 2023; Zheng et al. 2023). Hepatocellular carcinoma cells can also acquire drug resistance through modulation of mitophagy (Yao et al. 2022). In our study, we observed a significant enhancement of mitochondrial dynamics and concurrent p62 aggregation in cabozantinib-resistant hepatocellular carcinoma cells, which facilitated mitophagy. Moreover, in vivo experiments confirmed that inhibition of mitochondrial dynamics could enhance the therapeutic efficacy of cabozantinib.

In tumor cells, p62 protein is essential in the process of autophagy. Recent studies have identified p62 phase separation as a critical factor in autophagy, providing a platform for autophagic initiation (Berkamp et al. 2021). It has also been found to promote mitophagy, thereby leading to chemotherapy resistance (Peng et al. 2021). These findings suggest that autophagy, including mitophagy, serves as an important self-protective pathway in tumor cells, and p62 phase separation establishes a favorable foundation for chemotherapy resistance. In cabozantinib-resistant hepatocellular carcinoma cells SNU-368 and SNU-739, we observed a significant increase in p62 protein aggregation. Furthermore, interventions targeting p62 dissociation demonstrated the reversal of drug resistance, suggesting that co-inhibition of p62 aggregation may enhance the efficacy of cabozantinib. Therefore, the development of effective interventions targeting p62 aggregation represents a promising direction for further research in the field of drug discovery.

Despite these findings, our study has several limitations. First, the conclusions were primarily derived from two HCC cell lines and hydrodynamic injection-induced primary liver cancer models, which may not fully represent the genetic and phenotypic heterogeneity of human HCC. Further validation using additional cell lines, patient-derived xenograft models, or clinical cohorts would strengthen the generalizability of our results. Second, while we demonstrated that c-Myc overexpression promotes p62 aggregation-mediated mitophagy, the precise molecular mechanisms linking c-Myc to p62 phase separation remain unclear. Detailed structural analyses of p62 aggregation dynamics under c-Myc regulation, such as liquid-liquid phase separation assays or mutagenesis studies, are warranted. Third, the role of the tumour microenvironment (TME) in cabozantinib resistance was not addressed. Components of the TME, including immune cells, cancer-associated fibroblasts, and extracellular matrix remodeling, are known to influence drug response and tumour adaptation. Our experimental models did not incorporate these stromal interactions, potentially overlooking TME-driven resistance mechanisms. Future studies using co-culture systems or spatially resolved transcriptomics could clarify these contributions. Fourth, the clinical translatability of combining cabozantinib with autophagy inhibitors requires careful evaluation of toxicity profiles, pharmacokinetic interactions, and potential off-target effects, which were not systematically addressed in this preclinical investigation. Finally, our focus on mitophagy-related pathways might have overlooked other resistance mechanisms potentially involving c-Myc’s well-documented roles in metabolic reprogramming, genomic instability, or immune evasion. Future studies should explore these parallel pathways and their interplay with the TME to develop comprehensive therapeutic strategies.

In summary, our study has revealed that hepatocellular carcinoma cells can protect cellular function and develop cabozantinib resistance by augmenting p62 aggregation and mitochondrial dynamics, leading to mitophagy. These findings provide valuable insights for the development of sensitizing agents to enhance the efficacy of cabozantinib treatment and offer a novel perspective on exploring the mechanisms underlying tumor cell resistance.

## Supplementary Information

Supplementary Material 1.

## Data Availability

No datasets were generated or analysed during the current study.
