# Supplementary material for: Overexpression of c-Myc triggers p62 aggregation-mediated mitochondrial mitophagy in cabozantinib resistance of hepatocellular carcinoma
Source: Mol Med. 2025 May 27;31:209. doi: 10.1186/s10020-025-01263-w (PMC12107842; doi:10.1186/s10020-025-01263-w)
Supplement: Supplementary file 1 — Supplementary Material 1. [file 10020_2025_1263_MOESM1_ESM.docx]

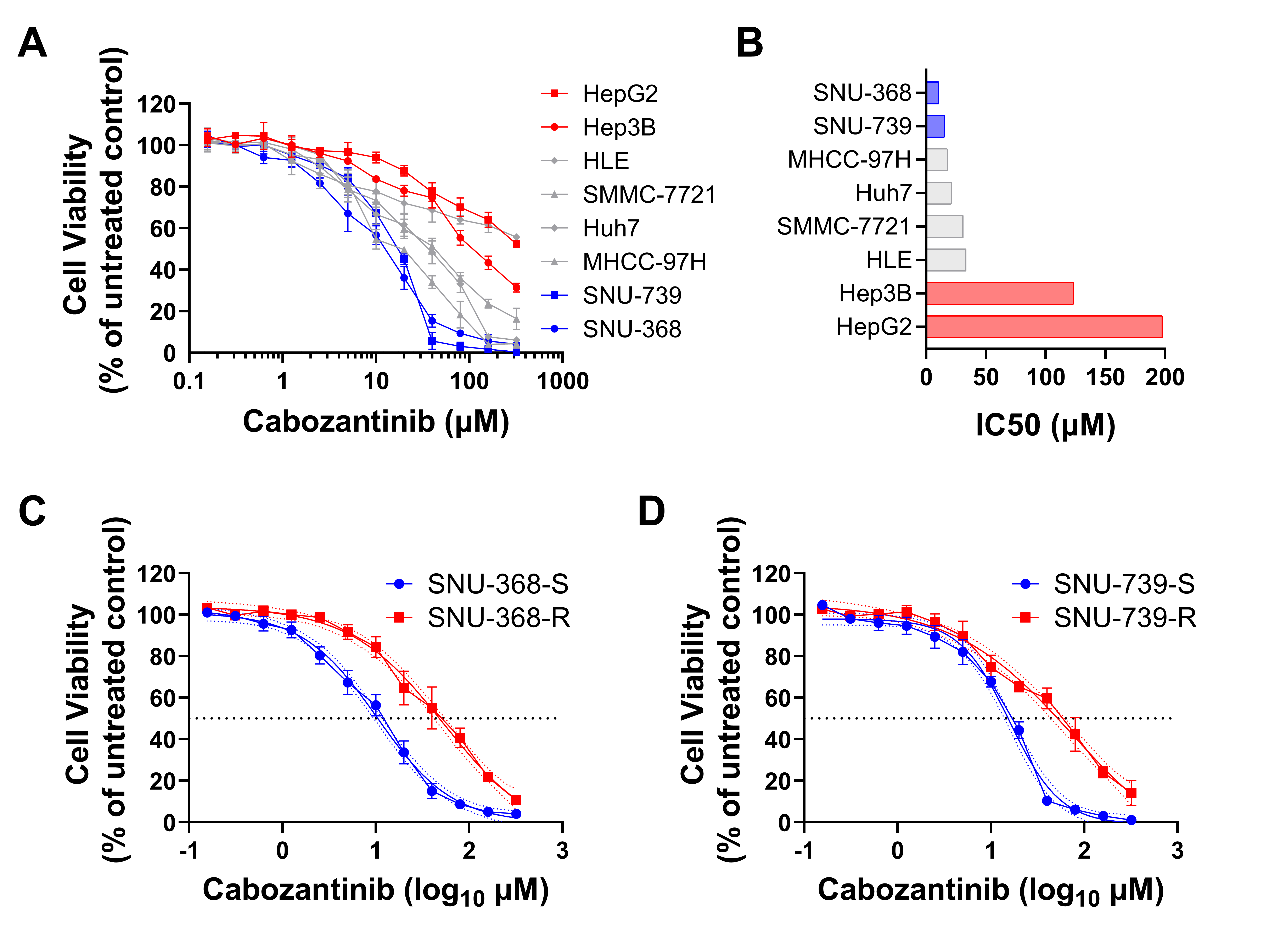


FigS1. Cabozantinib-Sensitive HCC Cell Lines Screening and Acquired Resistance Induction. A) Cell viability (CCK8) of various HCC cell lines in our center following cabozantinib treatment. B) IC50 values (CCK8) of HCC cell lines to cabozantinib, showing the lowest IC50 for SNU-368 and SNU-739. C) IC50 values (CCK8) before and after induction of acquired cabozantinib resistance in SNU-368 cell line. D) IC50 values (CCK8) before and after induction of acquired cabozantinib resistance in SNU-739 cell line.


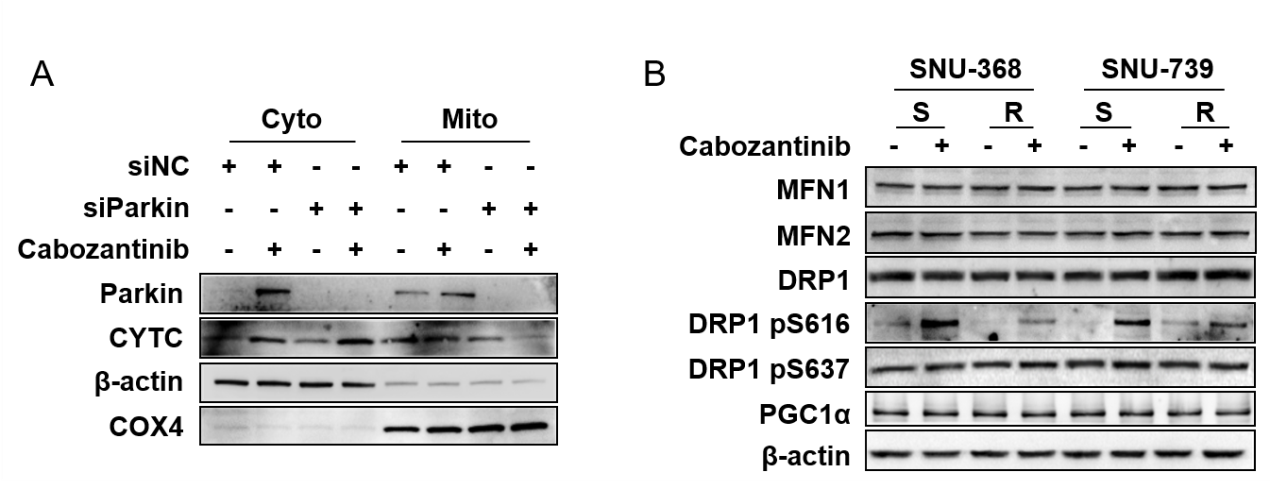


FigS2. Western Blot Analysis of Parkin, CYTC, MFN1/2, DRP1, DRP1 pS637, DRP1 pS616 and PCG1α.


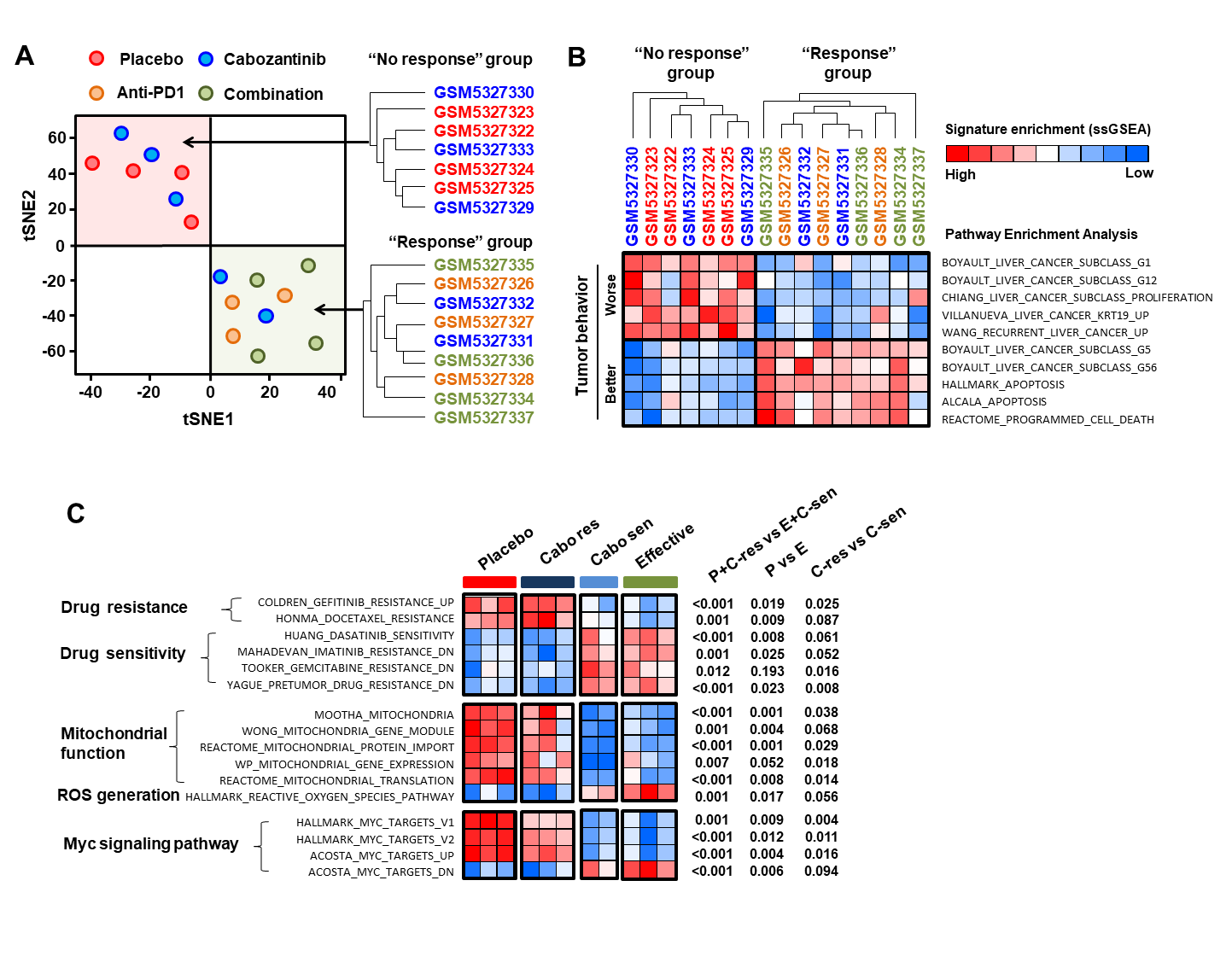


FigS3. Screening for Cabozantinib Resistance-Related Pathways (GSE174770). A) Following PCA, the sample clustering of the "No response" and "Response" groups revealed 3 samples resistant to cabozantinib and 2 samples sensitive to cabozantinib. B) Differential pathways following pathway enrichment analysis of the "No response" and "Response" groups. C) Pathway enrichment analysis of three cabozantinib-resistant samples and two cabozantinib-sensitive samples revealed significant differences in mitochondrial function-related pathways, ROS generation-related pathways, and the c-Myc signaling pathway.


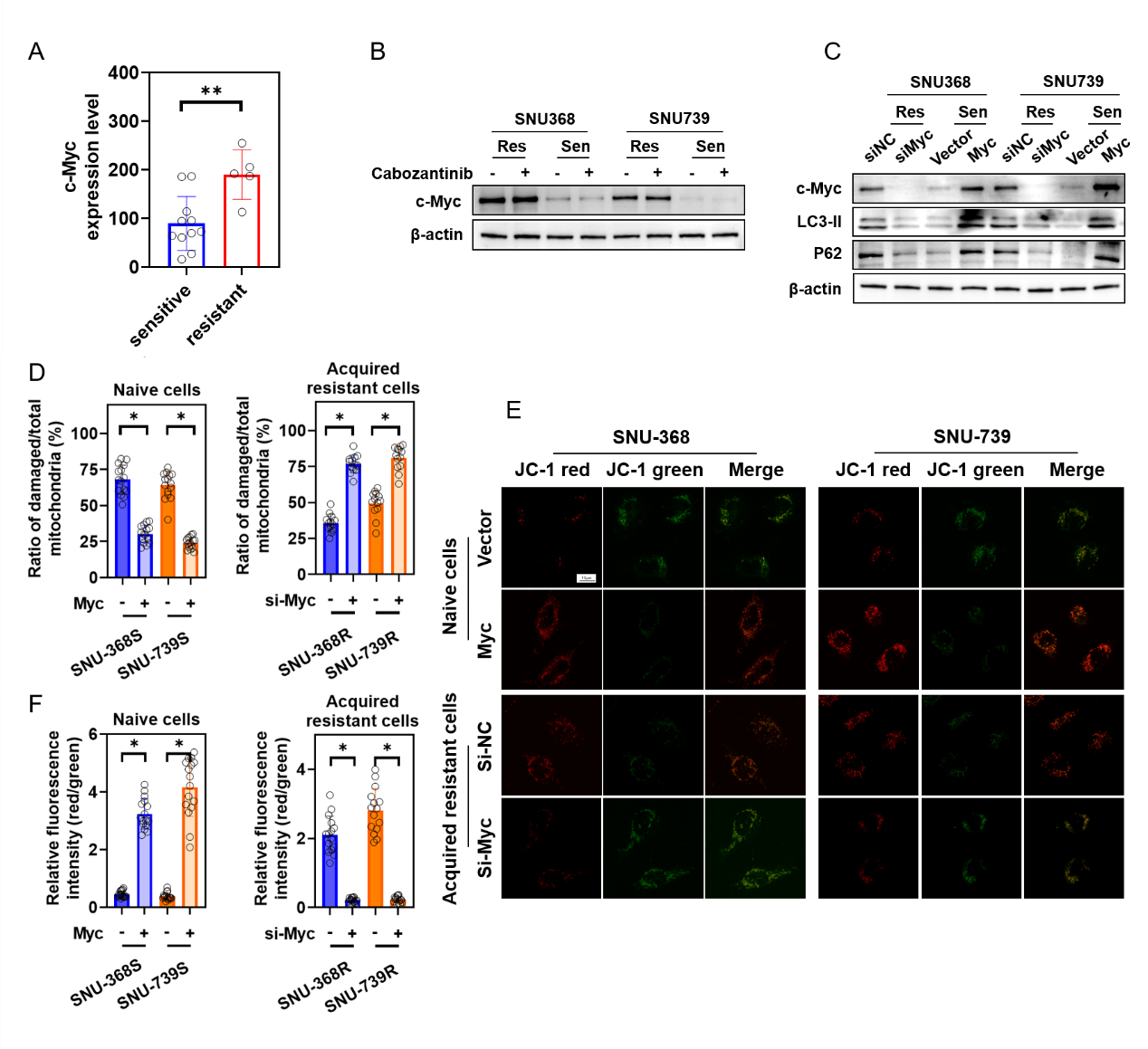


FigS4. High Expression of c-Myc Correlates with Cabozantinib Resistance in Liver Cancer Cells. A) c-Myc expression levels between sensitive and resistant HCC cell lines (GSE97098). B-C) Western blot analysis of c-Myc, LC3II and p62. D) Statistical analysis of the ratio of damaged mitochondria to total mitochondria. E-F) JC-1 analysis of mitochondrial membrane potential and statistical analysis of red/green fluorescence ratio for evaluating mitochondrial health status.


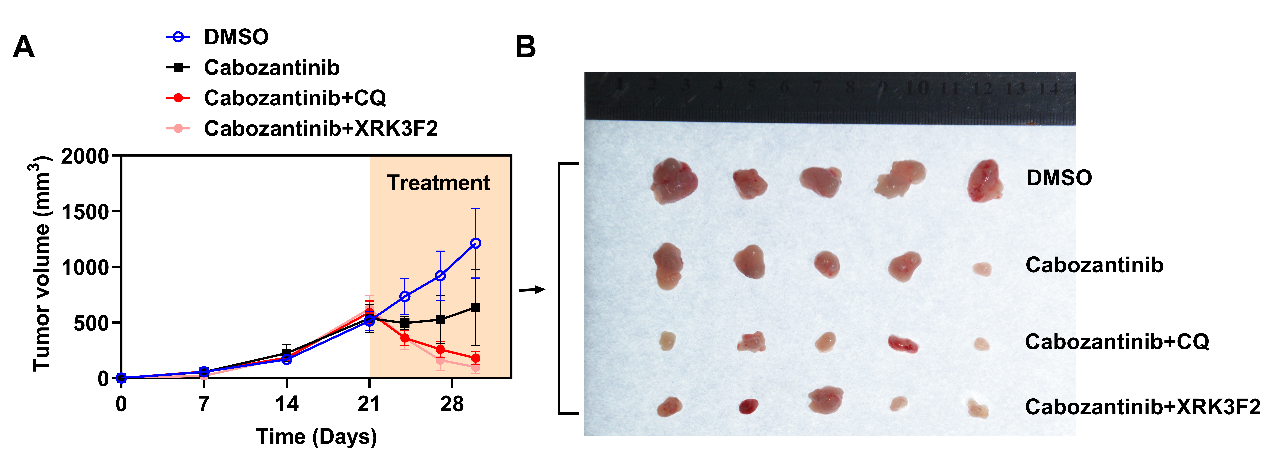


FigS5. Mice Subcutaneous Tumor Response to Cabozantinib and Combination Therapy. A) Tumor volume changes over time, showing significantly enhanced efficacy of cabozantinib after combination therapy. B) Comparison of subcutaneous tumor volumes among the different groups of mice.
